# Supplementary material for: Patterns of Intron Gain and Loss in Fungi
Source: PLoS Biol. 2004 Nov 30;2(12):e422. doi: 10.1371/journal.pbio.0020422 (PMC532390; doi:10.1371/journal.pbio.0020422)
Supplement: Table S1 — Also available at http://genes.mit.edu/NielsenEtAl/. (4.3 MB ZIP). [file pbio.0020422.st001.zip › NielsenEtAl/html/1355.html]

AN0764.1.NCU01979.1.MG02619.1.FG11347.1


```
 CLUSTAL W (1.82) Multiple Sequence Alignments - Introns Inserted


Sequence 1: AN0764.1	329 aa
Sequence 2: FG11347.1	326 aa
Sequence 3: NCU01979.1	253 aa
Sequence 4: MG02619.1	338 aa
Alignment Length: 343 aa
Number Identitical Residues: 123 aa
Alignment Score (without introns) 7281


MG02619.1 	MASQSTPG----GRGAASSSTPELLDLTIDNITQNTNVINSQAPSARLRYVMERLVTHLH
NCU01979.1	MAGTTTTTNTSTGNTNPTANLPPLLDLTIDNITPNTIRINSQASDARLKYLMARLVTHLH
FG11347.1 	-------------MAAP----VNLKDLTIDNITENVHAINSQCSSLRLKYILERVVTHLH
AN0764.1  	-------------MDPSQVKIPPMKNLTVENITENVIRINSLCEDERMKYVLERLVTHLH
          	                .  .   : :**::*** *.  *** . . *::*:: *:*****

MG02619.1 	DFARETRLSTEEWMAAIKFLIATGQISDDKRN~EFILLSDVLGLSLLVDNVNHPTPPGCT
NCU01979.1	DFARETRLSSDEWMAALQFLISCGKICSDVRN0EFILLSDVLGLSLLVDNINHPTLPGGT
FG11347.1 	DLARETRLTTDEWMTAIQFLTQVGQICTDVRQ0EFILLSDILGLSLLVDSIDHPKPKGST
AN0764.1  	DFARETRLSSAEWMTGLKFLTEVGQICTDVRQ0EFILLSDVLGLSILVDSIDHPKPPGST
          	*:******:: ***:.::**   *:*. * *: *******:****:***.::**.  * *

MG02619.1 	EGSVLGPFHTHDAPTLANGESMTSDPEGEPMLAVCTVKDRDGNPLAGVKVDIWETDSGGH
NCU01979.1	EGSVLGPFHTHDAPVLPNGSSMTSDPEGEPMLALCTVKDLKGNALPGVTIDIWETDSSGH
FG11347.1 	EGTVLGPFHTHEAEHVGEGSLISHDPDGEPLLVLCTVKDINGSPIDGASIDVWETDSKGF
AN0764.1  	EGTVLGPFHTHEAEELSQGGLMSKDPAGEPLLVVCTIKDTDGKPIEGVKVDIWETDSTGH
          	**:********:*  : :*  :: ** ***:*.:**:** .*..: *..:*:***** *.

MG02619.1 	YDVQHRDKDGPSERCVMISDDQGRFWFRGIKPVSYPIPNDGPVGKLLERLGRHCWRPAHV
NCU01979.1	YDVQHADRDAPSERCIMKSDDEGRFWFKGIKPVSYPIPHDGPVGKLLERLNRHPWRPAHV
FG11347.1 	YDVQHADRNGPDGRAVLKSDSEGNFWFKAIVPVPYPIPHDGPVGKLLKVLGRHPYRPSHM
AN0764.1  	YDVQHADRSGPDGRCIMHSNKDGVFWFNAITPVPYPIPHDGPVGKLLKKLHRHPYRPSHM
          	***** *:..*. *.:: *:.:* ***..* **.****:********: * ** :**:*:

MG02619.1 	HFMFEKEGWEKLIT2ALYLRGDPYEKTDAVFGVKSSLTIDLD-KVDAATSSEYGVPEGTW
NCU01979.1	HFKFEKDGWDPLVT~---------------------------------------------
FG11347.1 	HFMFKKDGYDPLIT2ALYLKDDPYESTDAVFGVKDSLVVSIQKVTDEEMAKKYDVKVGSA
AN0764.1  	HFMFEKQGYDHLIT~ALYLRNDPFETSDAVFGVKDSLVIDIG-TAGKEIAEKYGVEEGHK
          	** *:*:*:: *:* :    ... ..:.:  . ..:   .     .   :.. .   .  

MG02619.1 	LLRRDFVLVTEEETSKLRDDKAVEALKKLGLKVRLEDHLPVPDLD
NCU01979.1	---------------------------------------------
FG11347.1 	LMTYDFVLVTEKAAAELRRKKAEEAMAGLGRSFKFIDDLPVPDVD
AN0764.1  	LLTYDFVLVSEKETSELRERNSKEALDRLGRKVKIVNGLPIPDLD
          	    .    :.. ::.    .: .:    . .    .  . .. .
```
